# Caprylic Triglyceride as a Novel Therapeutic Approach to Effectively Improve the Performance and Attenuate the Symptoms Due to the Motor Neuron Loss in ALS Disease

**DOI:** 10.1371/journal.pone.0049191

**Published:** 2012-11-07

**Authors:** Wei Zhao, Merina Varghese, Prashant Vempati, Anastasiya Dzhun, Alice Cheng, Jun Wang, Dale Lange, Amanda Bilski, Irene Faravelli, Giulio Maria Pasinetti

**Affiliations:** 1 Department of Neurology, Mount Sinai School of Medicine, New York, New York, United States of America; 2 GRECC, James J Peters Veterans Affairs Medical Center, New York, New York, United States of America; 3 Hospital for Special Surgery, New York, New York, United States of America; Lousiana State University Health Sciences Center, United States of America

## Abstract

Amyotrophic lateral sclerosis (ALS) is a neurodegenerative disorder of motor neurons causing progressive muscle weakness, paralysis, and finally death. ALS patients suffer from asthenia and their progressive weakness negatively impacts quality of life, limiting their daily activities. They have impaired energy balance linked to lower activity of mitochondrial electron transport chain enzymes in ALS spinal cord, suggesting that improving mitochondrial function may present a therapeutic approach for ALS. When fed a ketogenic diet, the G93A ALS mouse shows a significant increase in serum ketones as well as a significantly slower progression of weakness and lower mortality rate. In this study, we treated SOD1-G93A mice with caprylic triglyceride, a medium chain triglyceride that is metabolized into ketone bodies and can serve as an alternate energy substrate for neuronal metabolism. Treatment with caprylic triglyceride attenuated progression of weakness and protected spinal cord motor neuron loss in SOD1-G93A transgenic animals, significantly improving their performance even though there was no significant benefit regarding the survival of the ALS transgenic animals. We found that caprylic triglyceride significantly promoted the mitochondrial oxygen consumption rate *in vivo*. Our results demonstrated that caprylic triglyceride alleviates ALS-type motor impairment through restoration of energy metabolism in SOD1-G93A ALS mice, especially during the overt stage of the disease. These data indicate the feasibility of using caprylic acid as an easily administered treatment with a high impact on the quality of life of ALS patients.

## Introduction

Amyotrophic Lateral Sclerosis (ALS) is a relentlessly progressive neurodegenerative disease causing weakness in skeletal muscles of the limbs, respiration and swallowing. It is invariably fatal and is caused by the death of neurons responsible for voluntary muscle control in the spinal cord and brain [1]. Currently, there is no effective treatment for ALS, and existing pharmacological therapy is limited to one agent, riluzole, causing modest slowing of disease progression. Furthermore, since the causes of ALS are likely multi-factorial and have been demonstrated to involve both genetic and environmental factors, targets for treatment are not easily identifiable [2], [3]. A large portion of familial forms of ALS (FALS) have been linked to a mutation in the superoxide dismutase (SOD)1 gene [4], [5], and several mouse models that express disease-related mutant SOD1 develop motor neuron degeneration similar to that in humans [6], [7], [8]. Despite the limitations of the transgenic SOD1 animal models, they remain the best tools to investigate potential new human therapies [9].

Mitochondrial abnormalities in ALS can be seen in the spinal motor neurons of ALS patients [10], [11] and in mutant SOD1 transgenic mice [12], [13], [14]. Decreased activity of electron transport chain complex IV has been observed in the spinal cords [15], [16] and muscle of ALS patients [17], [18], in the spinal cords of SOD1-G93A transgenic mice [19], [20], [21], and in a motor neuron cell line expressing SOD1 mutant proteins [22]. Lower mitochondrial respiration with complex I substrates has also been found in sporadic ALS (SALS) muscle [18], [23]. Chronic mitochondrial inhibition induces selective motor neuron death in vitro, recapitulating some of the pathological aspects of ALS [24]. It is noteworthy that mitochondrial dysfunction has been observed in both FALS and SALS patients, the latter group constituting the vast majority of patients with ALS. Consistent with mitochondrial dysfunction mechanism posited for ALS, the protective effect of energy metabolism substrates, such as coenzyme Q [25] and creatine [26], against neuronal damage induced by excitotoxicity and mitochondrial inhibition has been reported both *in vitro* and *in vivo*. In recent clinical trials, the mitochondrial modulator dexpramipexole [27] is emerging as a promising candidate for ALS therapy [28].

Ketones (or ketonic bodies), consisting essentially of D-β-3 hydroxybutyrate (DBH) and acetoacetate are derived from fat catabolism in liver mitochondria and are inversely proportional to the levels of glucose in the blood [29]. Circulating DBH crosses the blood-brain barrier and enters mitochondria where it is metabolized to acetoacetate and converted to acetyl-CoA, which enters into the Krebs cycle [30]. Ketogenic diets are high fat, low carbohydrate diets that increase levels of circulating ketones. Protective effects of ketogenic diets have been demonstrated in several neurological disorders notably in epilepsy [31], [32], and in rodent models of Parkinson’s disease [33], pain and inflammation [34] and juvenile traumatic brain injury [35], [36], [37]. We have previously reported the beneficial role of a ketogenic diet in improving motor function and survival of SOD1-G93A transgenic mice [38].

Medium chain triglyceride diets were developed as a more palatable modification of the ketogenic diet [39]. Medium chain triglycerides are six to twelve carbon fatty acid esters of glycerol. They are highly ketogenic and due to their small size, they are hydrolyzed into free fatty acids in the intestine and rapidly absorbed (reviewed in [40]). Caprylic triglyceride is an eight carbon medium chain triglyceride, which is metabolized into ketone bodies that can serve as an alternate energy substrate for neuronal metabolism. Caprylic acid is the main constituent of the medium-chain triglyceride diet advocated for seizure therapy [41] and it has been demonstrated to cross the blood-brain barrier [42], to exert antiepileptic effects [43] and to increase the effectiveness of the anticonvulsant drug, valproic acid [44], in mouse models of seizure. Also known as fractionated coconut oil, caprylic triglyceride is widely used in many skin products due to its rapid penetration ability. It has been previously developed as a medical food to promote mitochondrial metabolism in Alzheimer’s disease [45].

In this study, we treated SOD1-G93A transgenic mice with caprylic triglyceride to test whether this medium chain triglyceride may alter the progression of the motor impairment through modulation of mitochondrial energy metabolism.

## Materials and Methods

### Ethics Statement

All procedures were approved by the Mount Sinai Institutional Animal Care and Use Committee (IACUC).

### Experimental Animals and Diets

Male SOD1-G93A mutant transgenic mice (stock #002297) were obtained from the Jackson Laboratory (Bar Harbor, ME). Only males were used because of the background and gender effects on survival in this mouse model of ALS [21]. Caprylic triglyceride in its liquid form was purchased from Sigma Aldrich (St. Louis, MO) and used for both *in vitro* and *in vivo* studies. At 50 days of age, SOD1-G93A animals or their wild type littermates were placed on solid diets containing 10% (w/w) caprylic triglyceride (caloric composition: fat 34%, carbohydrate 46%, protein 20%) or a control isocaloric diet (Research Diets, New Brunswick, NJ). Mice had access to food and water *ad libitum*. Body weight, food intake and motor function assessment were monitored weekly. The survival study endpoint was defined as meeting any one of the following conditions: no spontaneous breathing or movement for 60 seconds with no response to pain; the animal was unable to roll over to the normal position within 10 s following a push over; or complete hind limb paralysis.

### Assessment of Motor Function

Mice were tested on an accelerating rotarod (7650 Ugo Basile Biological Research Apparatus, Comerio, Italy) as previously described. In brief, mice are placed onto a grooved cylinder (facing away from the experimenter) rotating at a predetermined speed that incrementally increases to a maximal rotation at 300 s; the time maintained on the rod by each mouse (latency) is then recorded (300 s max). A diminishing latency indicates declining performance and at values of 0 s is suggestive of severe muscular weakness and impaired coordination. Mice were tested weekly, beginning at 60 days of age, until they could no longer perform the test. Before testing, mice underwent a 1 week training period wherein they were introduced to the apparatus and handled by the experimenter daily. Testing was conducted during the last 4 h of the day portion of the light cycle in an environment with minimal stimuli such as noise, movement, or changes in light or temperature.

### Glucose Tolerance Test (GTT)

Mice were fasted overnight in clean cages with free access to water in new clean bottles. The next morning each mouse was weighed, and a baseline fasted blood glucose measurement was taken by applying tail blood to a Contour Blood Glucose Monitoring System (Bayer). Each mouse was injected intraperitoneally with a filter-sterilized solution of 20% (w/v) D-glucose, with the size of the bolus determined by animal weight (2 mg glucose/g body weight). Blood glucose measurements were taken as described above for each animal at 15, 30, 60 and 120 min. The data were plotted as blood glucose concentration (mg/dL) over time (minutes).

### Blood Ketone, Corticosterone and Triglyceride Level Assessment

Blood ketone level was obtained at pre-symptomatic stage (9 weeks) and post-symptomatic stage (17 weeks) by applying tail blood to a Precision Xtra® Blood Ketone Monitoring System (Abbott Laboratories) following the manufacturer’s instructions. Plasma corticosterone level was determined using Corticosterone ELISA (Enzo Life Sciences). Free glycerol and total triglyceride levels in the plasma were measured using Serum Triglyceride Determination Kit (Sigma Aldrich).

**Figure 1:**
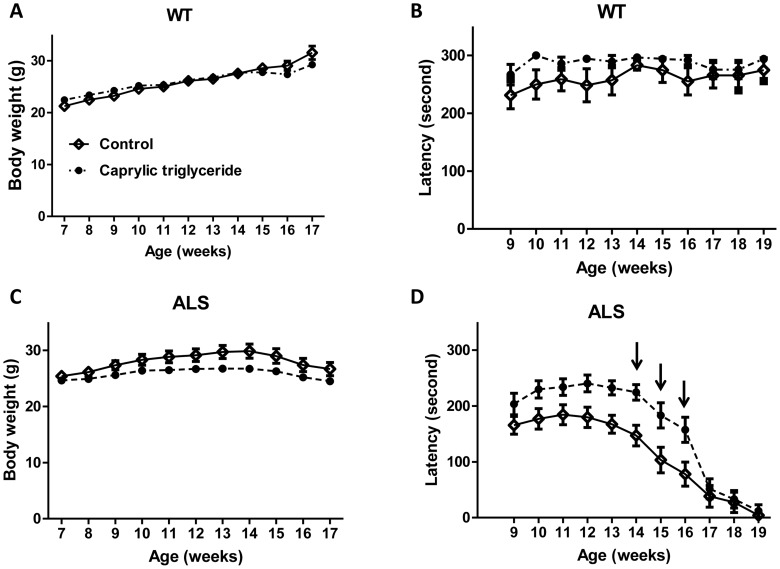
Caprylic triglyceride is well tolerated and attenuated ALS-type motor impairment in SOD1-G93A mouse model. (A) Average body weight in wild type (WT) animals (n = 6–7 per group); (B) Motor function as assessed by rotarod test in WT animals (n = 5–7 per group); (C) Average body weight in SOD1-G93A (ALS) animals (n = 17–18 per group); (D) Motor function as assessed by rotarod test in SOD1-G93A animals (n = 13–14 per group). Arrows indicate where the caprylic triglyceride group showed improved motor function as compared to the control by two way ANOVA analysis, F = 6.56, p = 0.01 followed by Bonferroni post-test, *p<0.05. All data are mean ± SEM.

### Motor Neuron Count in Lumbar Spinal Cord

Control and caprylic triglyceride treated mice were sacrificed at 110 days of age, when the motor deficits were apparent. The mice were anesthetized and perfused with phosphate buffered saline, followed by 4% para-formaldehyde. The lumbar spinal cord was dissected out and post-fixed in 4% para-formaldehyde. Spinal cords were embedded in paraffin and sectioned using a microtome to give 15 µm serial sections spaced 230 µm apart. The sections were stained with cresyl violet (Sigma Aldrich) using the following procedure: deparaffinization in Histo-Clear II (3×10 min), re-hydration in graded ethanol series 100% ethanol (5 min), 90% ethanol (3 min), 70% ethanol (3 min), 50% ethanol (3 min), rinse in tap water and then distilled water, staining in 0.1% Cresyl Violet (w/v) in 0.3% (v/v) glacial acetic acid (30 min at 50°C), rinse in distilled water, differentiation in series 50% ethanol (3 s), 70% acetic acid in ethanol (3 s), 95% ethanol (3 s), 100% ethanol (3 s), clearing in xylene (2 times), air drying (5 min) and mounting in Paramount media. Mosaic image acquisition covering the entire area of each section was performed under the 40×oil immersion objective of an Axioplan 2IE microscope with a motorized stage (Carl Zeiss Microscopy LLC, Thornwood, USA), using a Zeiss AxioCam MRc digital camera. The images from each section were stitched together using the Axiovision software. Neurons in the ventral horn were counted and were identified as large (>25 µm) cells having a pale nucleus with a distinct nucleolus and dark Nissl bodies in the cytoplasm [46], [47]. The total number of neurons in serial sections was compared between control and treated mice.

**Figure 2:**
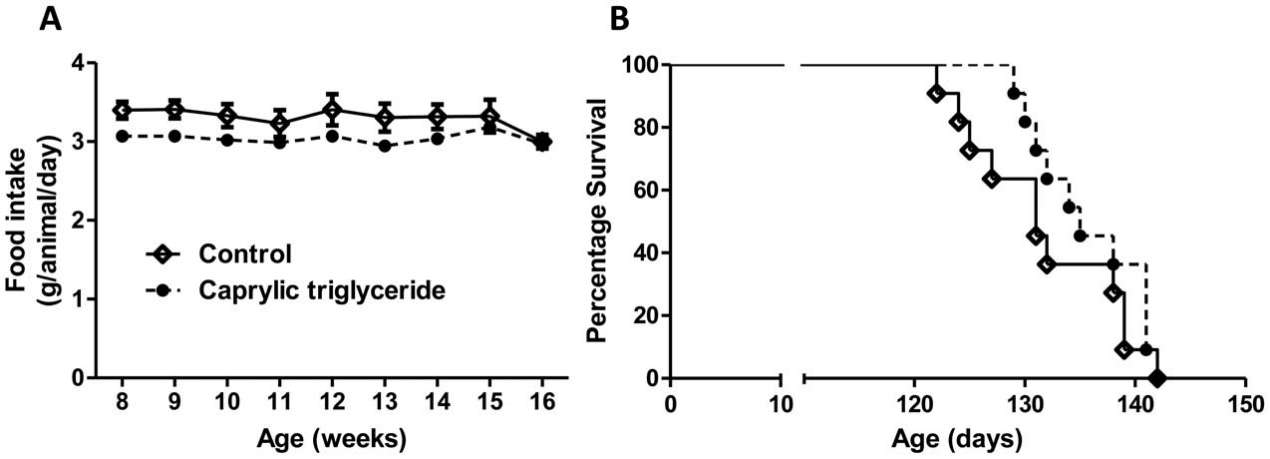
Effect of Caprylic triglyceride on food intake and lifespan of SOD1-G93A animals. (A) Food intake in SOD1-G93A animals treated with caprylic triglyceride (n = 18) or an isocaloric control diet (n = 17); (B) Mice in the two treatment groups (n = 11) were monitored daily and survival curve was plotted in GraphPad Prism.

### Mitochondrial Respiration in Spinal Cord

SOD1 G93A mice on control and caprylic triglyceride diet were sacrificed by carbon dioxide inhalation at 110 days of age (post-symptomatic stage). The spinal cords were dissected out and immediately processed for isolation of mitochondria. Mitochondria were prepared by differential centrifugation and mitochondrial respiratory function was measured using the Seahorse XF24 Extracellular Flux Analyzer (Seahorse Bioscience, Billerica, MA) as previously published [48], with the modification that 40 µg mitochondrial protein per well was used for complex I OCR and 20 µg for complex II OCR measurements. Spare respiratory capacity was calculated as the difference between the maximal and basal OCRs.

## Results

### Caprylic Triglyceride Significantly Attenuated ALS-type Motor Impairment in SOD1-G93A Transgenic Mice

Caprylic triglyceride did not result in any change in body weight (Fig. 1A) or motor performance (Fig. 1B) in wild type animals as compared to control group, suggesting that caprylic triglyceride is very well tolerated.

Important behavioral and physiological characteristics of SOD1-G93A transgenic mice include impaired motor performance, weight loss and reduced survival. SOD1-G93A mice fed caprylic acid showed no significant difference in weight as compared to those on control diet (Fig. 1C). There was no difference between the two groups in the amount of food consumed (Fig. 2A). In the caprylic triglyceride fed animals, motor function was significantly improved relative to controls (two way ANOVA analysis, F = 6.56, p = 0.01) (Fig. 1D), especially at week 14, 15 and 16 (Bonferroni post-test, *p<0.05).

We also monitored the survival of the experimental animals (Fig. 2B). SOD1-G93A animals on caprylic triglyceride diet had a median survival of 135 days. Although it was longer than the median survival of SOD1-G93A animals on control diet (129 days), it did not reach statistical significance (Mantel-Cox test, p = 0.165).

**Figure 3:**
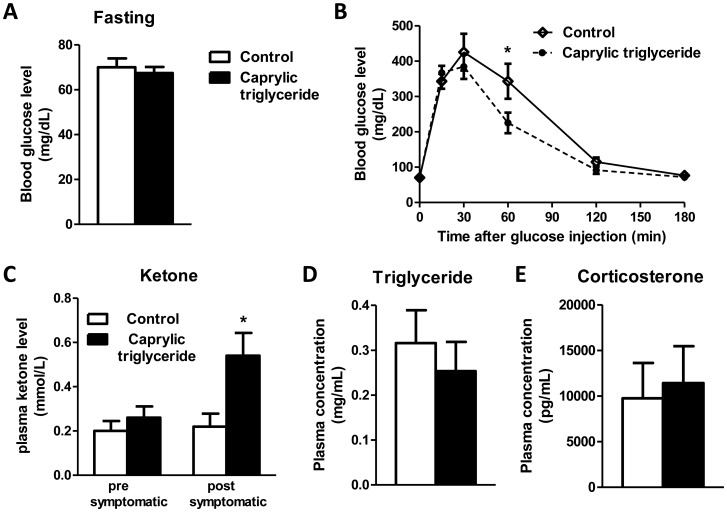
Glucose tolerance, ketone, triglyceride and corticosterone levels following caprylic triglyceride treatment. (A) Fasting serum glucose level and (B) glucose tolerance test in SOD1-G93A animals on control or caprylic triglyceride; (C) Blood ketone level at pre-symptomatic (10 weeks) or post-symptomatic (17 weeks) stage; (D) Plasma total triglyceride levels. (E) Plasma corticosterone level. All data are mean ± SEM, n = 4–5 for (A, B), n = 5 for (C) and n = 6–7 for (D, E) *p<0.05 by two-tailed t-test).

**Figure 4:**
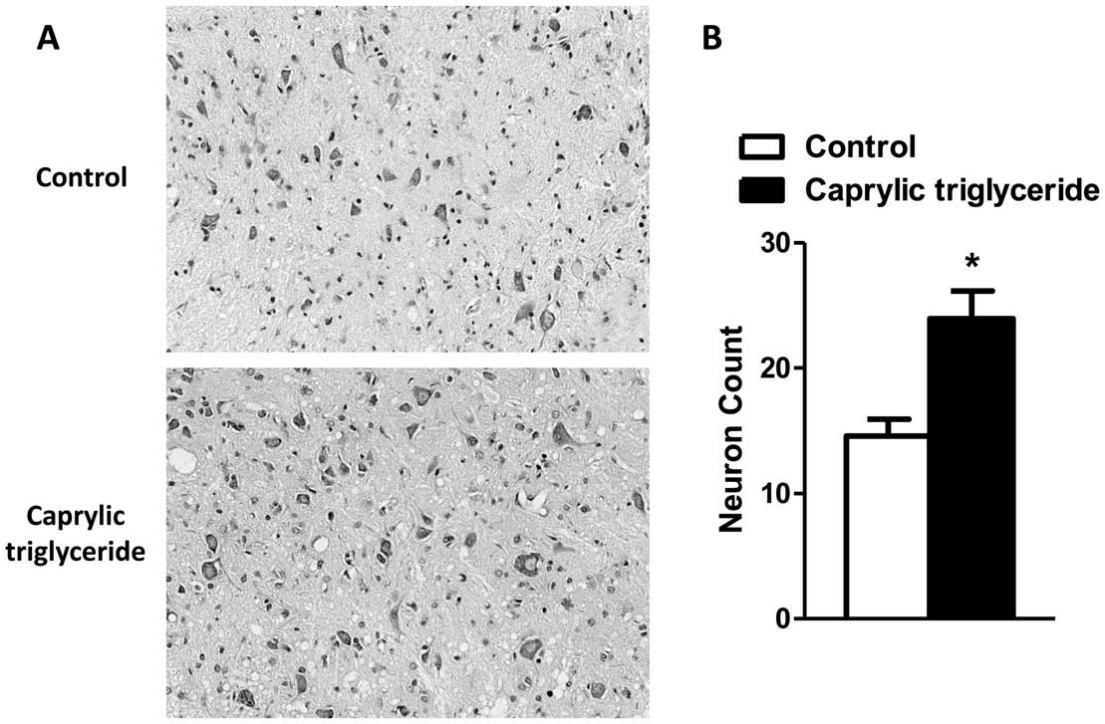
Nissl-stained motor neuron count in the lumbar spinal cord. (A) Representative photomicrographs of Nissl-stained sections at the ventral horn area of the lumbar spinal cord; (B) Motor neuron counts. Data are mean ± SEM, n = 3–4 per group, *p<0.05 by two-tailed t-test.

### Effect of Caprylic Triglyceride on Glucose Tolerance

Impaired glucose tolerance has been reported in ALS patients [49]. To test whether caprylic triglyceride could beneficially affect the regulation of blood glucose levels, we performed a glucose tolerance test in the experimental animals. We first compared the fasting blood glucose levels between the two groups (Fig. 3A) and found that caprylic triglyceride treatment did not change the fasting glucose level in SOD1-G93A animals. We also recorded their glucose levels at 15, 30, 60, and 120 min after glucose injection (2 mg/g body weight). Although two way ANOVA analysis did not reveal any major difference between the caprylic triglyceride treatment group and control group (Fig. 3B), a significantly lower glucose level at 60 min post-injection was observed in the animals fed with caprylic triglyceride (Bonferroni post-test, *p<0.05).

**Figure 5:**
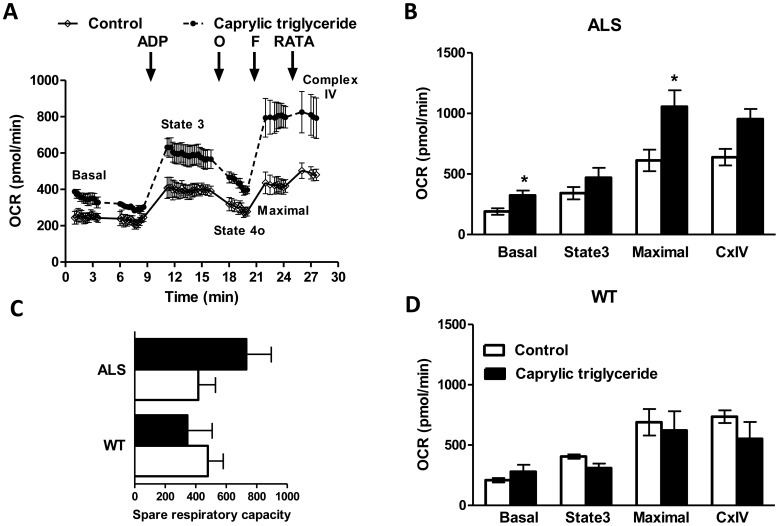
Mitochondrial bioenergetic profile in the spinal cord of WT and SOD1 G93A mice on control or caprylic triglyceride diet. Mitochondria were isolated by differential centrifugation from the whole spinal cord of SOD1-G93A mice on control or caprylic triglyceride diet and oxygen consumption rates were analyzed using Seahorse XF24 extracellular flux analyzer. (A) A representative trace of OCR in the presence of pyruvate and malate. Adenosine diphosphate (ADP), oligomycin (O), carbonyl cyanide 4-(trifluoromethoxy)phenylhydrazone (FCCP) and a mixture of rotenone, antimycin A, N,N,N’,N’-tetramethylphenylenediamine and ascorbate (RATA) were injected at the indicated time points to measure basal, state 3, state 4o, maximal and complex IV OCR as indicated. OCR in the presence of pyruvate and malate in (B) SOD1-G93A (ALS) and (D) wild type (WT) mice. (C) Spare respiratory capacity of mitochondria from WT and ALS mice on control or caprylic triglyceride diet. Data are mean ± SEM, n = 3 for all groups, *p<0.05 as compared to control by two-tailed student t-test.

### Caprylic Triglyceride Safely Increased Blood Ketone Level

Caprylic triglyceride is metabolized into ketone bodies in the liver. To determine whether caprylic triglyceride treatment could result in increased concentrations of ketone in the SOD1-G93A animals, we measured the blood ketone level in mice fed caprylic triglyceride or control isocaloric diet. The caprylic triglyceride fed mice showed about a 2.5 fold increase in the blood concentration of circulating ketones compared to animals on control diet (0.54±0.10 vs 0.22±0.06 mmol/L, p = 0.027 by two-tailed t-test) at post-symptomatic stage (Figure 3C). We also measured the lipid profile (Figure 3D) in the plasma and no difference was detected. Higher corticosterone levels have been demonstrated to significantly correlate with an earlier onset of paralysis in ALS patients [50]. We measured cortisosterone levels in the plasma (Figure 3E) and no difference was found following caprylic triglyceride administration.

### Caprylic Triglyceride Protected Against Motor Neuron Loss

To determine whether caprylic triglyceride can protect against the motor neuron loss that accompanies the clinical symptoms of ALS, we counted the number of motor neurons in the lumbar spinal cord in mice on the caprylic triglyceride diet compared to those on control isocaloric diet at the post-symptomatic stage (day 110) (Figure 4A). There were significantly higher numbers of motor neurons in the lumbar spinal cord of mice on a caprylic triglyceride diet than those on control diet (23.96±4.38 vs. 14.61±2.31, p = 0.02 by two-tailed t-test) (Figure 4B) and the treatment returned the motor neuron count to that found in WT mice of the same age (20.4±0.5; from our previously published data [51]).

### Caprylic Triglyceride Promoted Oxygen Consumption in Spinal Cord Mitochondria of SOD1-G93A Mice

To evaluate the effect of caprylic triglyceride in the mitochondrial bioenergetic profile, we isolated the spinal cord mitochondria of WT and SOD1-G93A animals fed 10% caprylic triglyceride or control isocaloric diet at post-symptomatic stage (day 110) and measured oxygen consumption rate using the Seahorse XF24 extracellular flux analyzer. WT mice on control and caprylic triglyceride diets had comparable OCRs (Fig. 5D). We found that basal and FCCP-induced maximal mitochondrial oxygen consumption rates in the presence of the complex I substrates pyruvate and malate were increased in the spinal cord of caprylic triglyceride treated SOD1-G93A mice relative to mice fed control isocaloric diet (Fig. 5A and 5B; p<0.05 by two-tailed t test). Following caprylic triglyceride treatment, spare respiratory capacity remained unchanged in WT mice and showed a trend for increase in SOD1-G93A mice which did not reach statistical significance (Fig. 5C). ADP-stimulated state 3 respiration and OCR with complex IV electron donors showed a trend for increase, which did not reach statistical significance (Fig. 5B). Oligomycin inhibited state 4o OCR remained unchanged (data not shown). There was no significant difference in OCR with the complex II substrate succinate in spinal cord mitochondria of WT as compared to SOD1 G93A mice or of the control and caprylic triglyceride SOD1 G93A mice (data not shown). This evidence suggested that long term treatment of caprylic triglyceride changed the metabolic response, possibly through alterations of the checkpoints for control of mitochondrial respiration.

## Discussion

Our study demonstrated that SOD1-G93A transgenic animals fed caprylic triglyceride showed significant improvement in the clinical signs of ALS and in motor neuron survival in the spinal cord. The improvement of motor performance in caprylic triglyceride-treated animals (Fig. 1D) was accompanied by significantly more motor neurons preserved in the spinal cord at the end stage of disease (Fig. 4). These findings are similar to our previously reported findings in G93A transgenic mice fed a ketogenic diet [38] as well as the R6/2 1J Huntington’s disease model [52].

It has been reported that ALS patients with elevated triglyceride and cholesterol serum levels have a prolonged survival [53]. Our data showed that animals treated with caprylic triglyceride did not have a prolonged life span as compared to the control group, possibly due to the fact that the treatment did not result in increased serum triglyceride/cholesterol level (Fig. 3D). Future studies will address the effect, if any, of caprylic triglyceride on denervation in the neuromuscular junction, which may be a contributory factor to the lack of effect on survival following treatment. It should be noted that the transgenic mouse and the treatment used in this study were quite stringent–a severe genetic model and a highly restrictive diet formulation. It will also be necessary to assess the efficacy of caprylic triglyceride diet in other models of ALS such as the TDP-43 or FUS/TLS mice to determine whether the benefits we have observed are due to specific to the SOD1 mutant.

Caprylic triglyceride can be metabolized into ketone bodies, and serve as an alternate energy substrate for neuronal metabolism. Our results showed increased basal and maximal mitochondrial oxygen consumption rates in the spinal cord together with elevated ketone levels in the blood of caprylic triglyceride-treated ALS transgenic animals, suggesting that the additional ketones provided by caprylic triglyceride might be used as an alternate substrate for energy metabolism in ALS animals. The increased maximal respiration (Fig. 5C) may represent improved mitochondrial respiratory capacity in the disease model. Further studies are required to determine whether the trend for increase in mitochondrial respiratory capacity is due to changes in the neuronal or glial mitochondrial content and/or regulation of metabolic activities. It is noteworthy that the decrease in mitochondrial oxidative phosphorylation activity observed by histochemical methods in the spinal cord of SOD1-G93A mice as compared to WT [51] is not detectable by the assay used in this study (Fig. 5B and 5D). This could be due to the fact that the current assay using mitochondrial preparations from the whole spinal cord cannot detect the area-specific decrease in mitochondrial activity in the ventral horn of the lumbar spinal cord. Another drawback of our study is that the mice were sacrificed by carbon dioxide inhalation and exposure to hypercapnic/hypoxic conditions are known to alter mitochondrial function [54]. We have taken maximum care to ensure that the different groups of mice were treated identically during euthanasia but the effects of hypoxia cannot be completely discounted.

It has been reported that caprylic triglyceride, when used as a medical food, attenuates cognitive dysfunction in Alzheimer’s disease [55]. Although further research is needed to better understand the mechanism and magnitude of the clinical impact that caprylic acid has on transgenic mouse models, there is now a basis to move this treatment into human studies. The ability to objectively measure pharmacologic effects (ketone bodies and lipids, Fig. 3C and 3D) will easily translate into phase I safety and tolerability, accelerating the decision whether or not to proceed with efficacy studies.
